# The dual nature of *Fusarium oxysporum* Fo47: from plant disease suppression to potential pathogenicity risks

**DOI:** 10.3389/fmicb.2026.1920231

**Published:** 2026-10-01

**Authors:** Marta Burgberger, Stanisław Roguż, Beata Augustyniak, Wioleta Wojtasik

**Affiliations:** Department of Genetic Biochemistry, Faculty of Biotechnology, Wrocław University, Wrocław, Poland

**Keywords:** biocontrol, endophyte, *Fusarium oxysporum*, HGT, priming

## Abstract

Fungal diseases threaten crop productivity, and the limitations of intensive fungicide use highlight the need for sustainable plant protection strategies. The non-pathogenic, endophytic strain *Fusarium oxysporum* Fo47 is one of the best-characterized models of disease-suppressive fungi. This review summarizes current knowledge on Fo47 as a beneficial endophyte and biological control agent, while also considering biosafety concerns related to the horizontal transfer of pathogenicity determinants. Fo47-mediated protection involves direct mechanisms, including competition, antibiosis and physical interference with pathogens, as well as indirect plant-mediated responses such as endophyte-mediated resistance and priming. Fo47 colonization may modulate PR proteins, ROS and antioxidant metabolism, cell wall remodeling, secondary metabolism and hormone-related signaling. However, these responses depend on the host plant, pathogen species and timing of application. Therefore, Fo47 represents a valuable model for endophyte-mediated disease suppression, but its broader agricultural use requires careful risk assessment. This review focuses primarily on evidence from tomato, pepper and flax pathosystems, while also incorporating findings reported in several other plant hosts.

## 1 Introduction

Plant diseases, especially those caused by fungal pathogens, threaten global food security by reducing crop yield and quality. Fungal infections can cause 20%–30% losses in agricultural production, making them a major challenge for sustainable agriculture (Fisher et al., 2012; Rizzo et al., 2021; Savary et al., 2019). Although synthetic fungicides are widely used, their overuse raises concerns related to environmental pollution, biodiversity loss, residues, and pathogen resistance. Therefore, biological control using beneficial microorganisms is increasingly considered an important sustainable alternative in integrated pest management (Collinge et al., 2022; Villavicencio-Vásquez et al., 2025). Among these beneficial microorganisms, the endophytic, non-pathogenic strain *Fusarium oxysporum* Fo47 represents a promising biological control agent.

Fo47 has become one of the best-studied non-pathogenic *F. oxysporum* strains because of its ability to colonize plant roots without causing disease and to reduce the development of diseases caused by several soil-borne pathogens. Its protective activity is associated with a combination of direct interactions with pathogens and plant-mediated defense responses. Direct mechanisms may include competition for nutrients and root colonization sites, growth inhibition, antibiosis and, in some interactions, mycoparasitism. Fo47 colonization can modify the physiological and molecular state of the host, leading to activation or enhanced responsiveness of defense-related pathways (de Lamo and Takken, 2020; Fravel et al., 2003). These responses involve pathogenesis-related (PR) proteins, reactive oxygen species (ROS), structural and cell wall-associated defenses, and changes in primary and secondary metabolism, including polyamines and phytohormones. Prior colonization may also prime plants for a faster or stronger response to subsequent pathogen challenge (Aimé et al., 2013). However, the effectiveness and mechanisms of Fo47-mediated protection vary depending on the host plant, pathogen, inoculation strategy and experimental conditions. Understanding this variability is essential for determining when Fo47 can provide reliable disease suppression and how its direct and indirect modes of action interact.

Pathogenic and non-pathogenic strains can coexist in the same ecological niches, which is why the potential horizontal transfer of pathogenicity-associated determinants represents an important issue that should be considered when evaluating long-term biocontrol applications. Recent omics-based approaches provide increasingly powerful tools for distinguishing non-pathogenic from pathogenic isolates and for identifying genes and pathways involved in colonization, antagonism and host defense activation. Genome-editing technologies further offer opportunities to investigate the functions of candidate genes and, potentially, to improve beneficial traits in non-pathogenic fungi.

This review summarizes current knowledge of Fo47 as a model endophytic biocontrol agent, from its biological characteristics and colonization strategy to the direct and indirect mechanisms underlying disease suppression. The review also addresses the potential risks associated with horizontal transfer of pathogenicity determinants and considers how omics and genome-editing approaches may contribute to the characterization, safety assessment and future development of non-pathogenic *F. oxysporum* strains as biological control agents.

## 2 Fo47 as a model endophytic biocontrol agent - discovery, biological characteristics and biocontrol activity of Fo47

The *F. oxysporum* species complex comprises a genetically and functionally diverse group of globally distributed soilborne fungi, including both pathogenic and non-pathogenic strains (Alabouvette and Olivain, 2002; Fravel et al., 2003; Gordon, 2017; Iida et al., 2022; Ma et al., 2010). Pathogenic members are classified into more than 120 formae speciales (f.sp.), primarily according to their host specificity, and cause vascular wilt, root rot and other diseases in numerous economically important crops, including banana, cotton, date palm and flax (Michielse and Rep, 2009). Vascular wilt caused by *F. oxysporum* is among the most destructive crop diseases worldwide, and because effective curative treatments are generally unavailable once symptoms have developed, disease management relies mainly on preventive strategies such as resistant cultivars, crop rotation and biological control (Dean et al., 2012; Theron et al., 2023; Yadeta and Thomma, 2013). Infection usually begins with the germination of chlamydospores in the vicinity of host roots, followed by penetration through root tips, lateral root emergence sites, root hairs or wounds. The hyphae subsequently grow through the root cortex and enter the xylem, where hyphae and microconidia spread through the transpiration stream and progressively disrupt water and nutrient transport. This vascular colonization leads to stomatal closure, wilting and, in severe cases, plant death (Gordon, 2017; Pietro et al., 2003; Upasani et al., 2016). Pathogenicity is often associated with accessory chromosomes carrying virulence-related genes, including genes encoding Secreted in Xylem (SIX) effectors, although additional mechanisms may also contribute to disease development (Ma et al., 2010; Schmidt et al., 2013; van der Does et al., 2008). *F. oxysporum* species complex includes not only plant pathogens but also beneficial strains with potential applications in biological control and sustainable plant protection (Alabouvette and Olivain, 2002; Constantin et al., 2021; Fravel et al., 2003; Gordon, 2017; Iida et al., 2022).

Non-pathogenic endophytic fungi are plant-associated fungi that can colonize internal plant tissues without inducing visible disease symptoms. They may enter plants through root tips, lateral root emergence sites, wounds or natural openings and subsequently persist within host tissues. Such fungi can contribute to plant protection by competing with pathogens for nutrients and colonization sites, producing antimicrobial metabolites or lytic enzymes, and activating host defense responses. Their interactions with plants are therefore often beneficial and may improve plant resistance to both biotic and abiotic stresses (de Lamo and Takken, 2020; Fadiji and Babalola, 2020; Latz et al., 2018; Xia et al., 2022; Yadav, 2018). One of the best-characterized non-pathogenic strains of *F. oxysporum* is Fo47. This strain was originally isolated from suppressive soils in the Châteaurenard region of France, a long-established market-gardening area characterized by intensive vegetable production, where vascular wilt diseases occurred at unusually low frequencies despite the presence of pathogenic *F. oxysporum* populations. Early studies identified a high abundance of non-pathogenic *F. oxysporum* isolates in these soils, suggesting their involvement in natural disease suppression (Alabouvette, 1986; Alabouvette et al., 1985; Siegel-Hertz et al., 2018). Fo47 is an endophytic strain capable of colonizing root tissues without causing visible disease symptoms. Unlike pathogenic *F. oxysporum* strains, it does not induce vascular wilt or plant death, and colonized roots remain functional throughout the interaction (Alabouvette, 1986; Alabouvette et al., 1987). The presence of Fo47 has been associated with enhanced tolerance to both biotic and abiotic stresses and with reduced disease development in several plant species (Akram et al., 2023; de Lamo and Takken, 2020; Fravel et al., 2003; Latz et al., 2018).

Fo47 has become one of the most extensively studied non-pathogenic *F. oxysporum* strains and a model organism for investigations of endophyte-mediated disease suppression, owing to its stable endophytic lifestyle, strong colonization ability, and reproducible protective effects (de Lamo and Takken, 2020), which have been demonstrated in numerous plant species, including tomato (Constantin et al., 2020, 2019), cucumber (Park, 1988), flax (Duijff et al., 1999), asparagus (He and Wolyn, 2005) and chickpea (Kaur and Singh, 2007). Disease suppression has also been reported when pathogenic and non-pathogenic *F. oxysporum* strains were applied simultaneously, resulting in reduced symptom severity compared with plants inoculated with pathogenic strains alone (Huertas-González et al., 1999). Furthermore, Fo47-mediated protection extends beyond *F. oxysporum* pathosystems. Fo47 has been shown to suppress several root-infecting pathogens, including *Verticillium dahliae*, *Phytophthora capsici* and *Pythium ultimum*, while no protection was observed against the foliar pathogen *B. cinerea* in pepper. These observations suggest that Fo47-mediated protection is particularly effective against root-associated diseases and pathogens that invade through the root system (de Lamo and Takken, 2020; Veloso and Díaz, 2012).

## 3 Colonization strategy of non-pathogenic *Fusarium oxysporum*

Fo47 colonization begins with adhesion to the root surface, followed by spore germination and directional hyphal growth near or on the root surface (Mekapogu et al., 2021; Olivain et al., 2006). Then, hyphae grow directionally in response to root and fungal secretions, colonize the root surface, and branch after contact with root tissues and penetrate host tissues. Fo47 may colonize the root surface and cortex. Entry may occur through root tips, root hairs, wounds, epidermal discontinuities and intercellular spaces within root tissues (de Lamo and Takken, 2020; Gordon, 2017; Upasani et al., 2016). Following penetration, Fo47 establishes an endophytic association with the host and colonizes root tissues without inducing visible disease symptoms. Fo47 does not extensively invade the vascular system or induce vascular wilt. Colonized roots remain functional, allowing long-term coexistence between the host and the endophyte (Alabouvette, 1986; Alabouvette et al., 1987; Fravel et al., 2003). Fo47 is generally detected on the root surface and within the outer cortical layers, with limited progression toward the vascular tissues (Benhamou et al., 2002; Gordon, 2017). Although Fo47 can colonize plant roots, its growth within host tissues is usually more restricted than that of pathogenic *F. oxysporum* strains. The biomass of pathogenic strains in roots is generally higher than that of endophytic strains, especially during the early stages of interaction (Validov et al., 2011). The restricted colonization pattern of Fo47 may be partly related to differences in the ability to degrade plant cell walls. The establishment of a beneficial endophytic interaction therefore requires a sufficient, but spatially controlled, colonization of host tissues. In endophytic fungi, this process may depend less on the formation of highly specialized penetration structures and more on the regulated secretion of cell wall-modifying enzymes, which enable entry into root tissues without extensive host damage (Tian et al., 2026). Fungal cell wall-degrading enzymes facilitate penetration and spread within host tissues by modifying or breaking down structural components such as pectin, hemicellulose and cellulose (Ishida and Noutoshi, 2022; Sharma and Gautam, 2019). Among these enzymes, pectinases, xylanases and cellulases are particularly relevant during fungal colonization, as they target major polysaccharide components of the plant cell wall and middle lamella (Sharma and Gautam, 2019). Since extensive colonization of the root cortex and vascular system correlates with increased secretion of cell wall-degrading enzymes, this trait is considered characteristic of pathogenic strains (Jonkers et al., 2009). In line with this, Fo47 differs from pathogenic *F. oxysporum* strains not only in the amount of biomass produced in roots, but also in the depth and pattern of tissue colonization (Benhamou and Garand, 2001; Nahalkova et al., 2008). In a study comparing the *Arabidopsis thaliana* pathogen Fo5176, the tomato pathogen Fol4287 and the endophyte Fo47, both pathogenic strains encoded more plant cell wall-degrading enzymes than Fo47. For example, Fo47 encoded only one glycoside hydrolase, compared with 13 in Fol4287 and nine in Fo5176. Moreover, 20 enzymes encoded by Fo5176 lacked orthologs in Fo47 and were upregulated during plant infection at one or more time points (Martínez-Soto et al., 2023). These quantitative differences support the broader view that pathogenic strains possess a more extensive enzymatic capacity for overcoming structural barriers, whereas the reduced cell wall-degrading enzymes (CWDE) repertoire of Fo47 may contribute to its more spatially restricted and less destructive colonization pattern. Although vascular colonization is generally associated with pathogenic *F. oxysporum* strains, Fo47 has also been detected in vascular tissues under specific conditions. Fo47 was reported to colonize xylem vessels in eucalyptus (Salerno et al., 2000). Moreover, Fo47 hyphae were observed in the vasculature of *A. thaliana* and tomato roots, but this presence was restricted to lateral roots and the elongation zone of primary roots. In contrast, only pathogenic strains were able to reach xylem tissues above the primary-root maturation zone. These observations indicate that Fo47 may occasionally enter vascular tissues, but its colonization remains spatially limited compared with pathogenic strains (Martínez-Soto et al., 2023). Consistent with this, the presence of the non-pathogenic Fo47 strain was demonstrated not only in flax roots, but also in shoots, suggesting that Fo47 can move beyond the root colonization zone under flax experimental conditions (Burgberger et al., 2026b).

## 4 Plant defense responses associated with Fo47 colonization

Fo47 colonization is recognized by the plant as a microbial interaction, but it does not lead to typical disease development. Fungal molecules such as chitin and β-glucans may activate pattern-recognition receptors (PRR)-mediated pattern-triggered immunity (PTI)-like responses, including Ca^2 +^ influx, ROS production, MAPK activation, callose deposition and cell wall reinforcement (Chuberre et al., 2018; de Lamo and Takken, 2020; Latz et al., 2018). Unlike pathogenic *F. oxysporum* strains, which can suppress or evade host immunity through effectors, Fo47 appears to induce a controlled defense response compatible with endophytic colonization (de Lamo and Takken, 2020; van Dam et al., 2017b). A schematic overview of the main plant defense responses associated with Fo47 colonization is presented in Figure 1.

**FIGURE 1:**
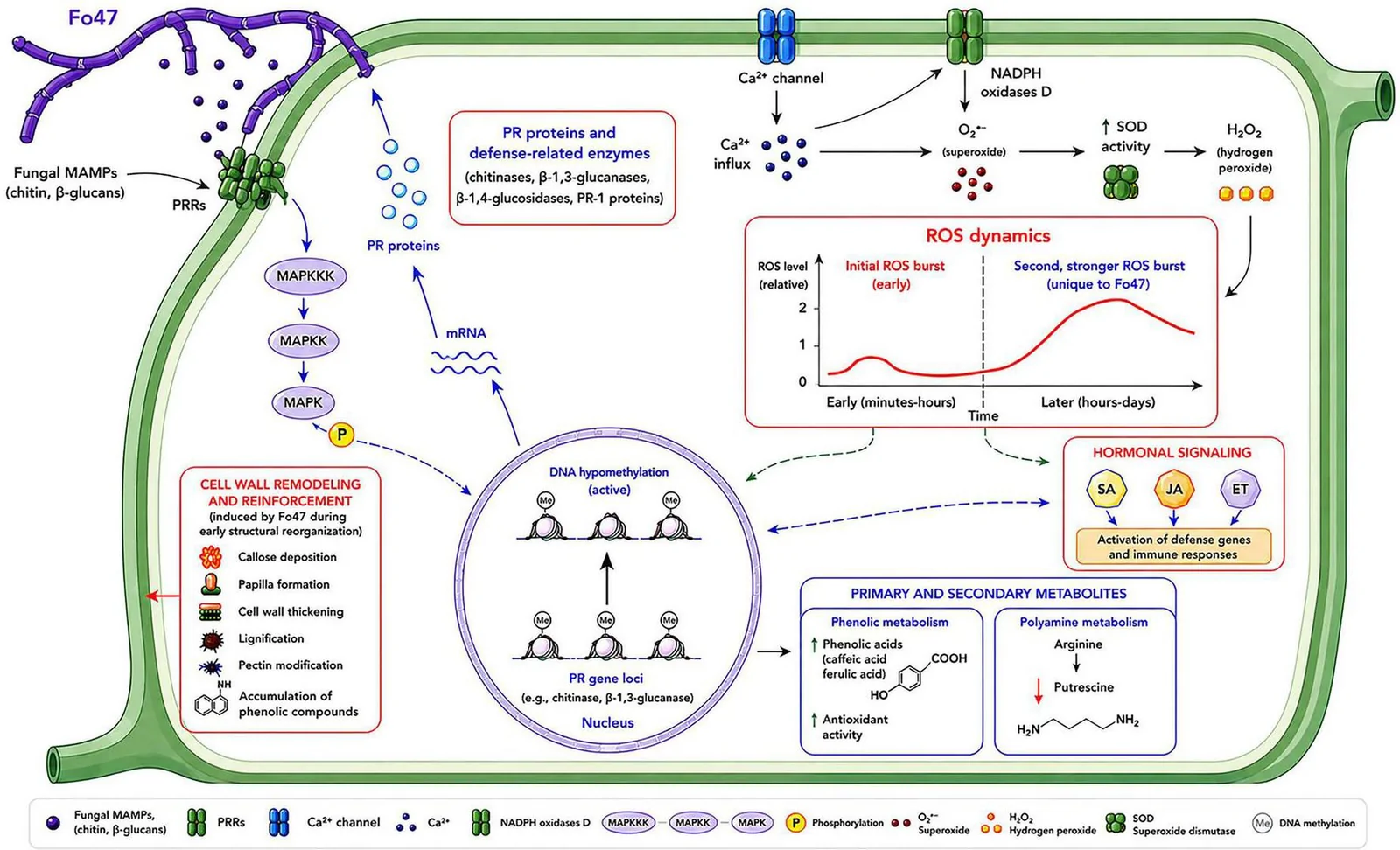
Plant defense responses associated with *Fusarium oxysporum* Fo47 colonization. Colonization of roots by the non-pathogenic endophyte Fo47 initiates a multilayered defense program. Fungal microbe-associated molecular patterns (MAMPs), including chitin and β-glucans, are recognized by plant pattern recognition receptors (PRRs), leading to early Ca^2 +^ influx, activation of MAPK signaling cascades, and an oxidative burst. Shortly after inoculation, Fo47 induces an initial ROS burst, followed by a second, stronger ROS burst that is unique to Fo47 colonization and is associated with activation of the antioxidant system. This response includes increased NADPH oxidase D expression, enhanced superoxide dismutase (SOD) activity, reduced superoxide (O_2_^•−^) accumulation, and controlled H_2_O_2_ production, creating a signaling environment that promotes defense activation while minimizing oxidative damage. Fo47-induced hypomethylation of chitinase and β-1,3-glucanase genes promotes the activation of PR proteins and other defense-related enzymes, including chitinases, β-1,3-glucanases, β-1,4-glucosidases, and PR-1 proteins. Simultaneously, Fo47 stimulates extensive cell wall remodeling and reinforcement, including cell wall thickening, papilla formation, callose deposition, lignification, accumulation of phenolic compounds, and early structural reorganization of the plant cell wall. In parallel, Fo47 modulates secondary metabolism by promoting the accumulation of defense-associated phenolics, such as caffeic and ferulic acids, and regulates polyamine metabolism through reduced putrescine accumulation. Fo47-mediated resistance also involves coordinated hormonal crosstalk among salicylic acid (SA), jasmonic acid (JA), ethylene (ET), and other signaling molecules rather than activation of a single canonical signaling pathway. Together, these interconnected local and systemic responses establish a primed physiological state that enhances resistance to subsequent pathogen infection while maintaining a beneficial endophytic association.

### 4.1 Pathogenesis-related proteins

Activation of PR proteins is among the most frequently reported responses associated with Fo47-mediated protection (Aimé et al., 2008; Fuchs et al., 1997; Veloso and Díaz, 2012). In tomato, Fo47 reduced disease caused by *F. oxysporum* f. sp. *lycopersici* even when the beneficial and pathogenic strains were applied separately in space or time. This protective effect was accompanied by enhanced activities of defense-related enzymes, including chitinase, β-1,3-glucanase and β-1,4-glucosidase (Fuchs et al., 1997) Also, Fo47 stimulated the accumulation of PR proteins including chitinases and PR-1 family proteins (Duijff et al., 1998; Fravel et al., 2003). Furthermore, in tomato plants pre-inoculated with Fo47 and subsequently challenged with Fol8, defense-related genes encoding chitinase, β-1,3-glucanase and PR-1a were overexpressed, which was accompanied by reduced pathogen colonization in the roots (Aimé et al., 2013). Similarly, transient induction of defense-associated genes such as *CABPR1* (encoding PR-1 protein), *CACHI2* (encoding class II chitinase), and *CASC1* (encoding sesquiterpene cyclase involved in the synthesis of capsidiol, a phytoalexin) was reported in pepper roots, with stronger activation occurring after pathogen challenge (Veloso and Díaz, 2012). However, constitutive activation of PR proteins is not always observed. In some systems, Fo47-colonized plants displayed lower expression of defense-related genes than pathogen-infected plants while still exhibiting disease protection (Aimé et al., 2008). These observations suggest that Fo47 may promote enhanced defense readiness rather than continuous activation of immune responses.

A more recent study on flax seedlings further demonstrated that Fo47 alone can induce PR-related gene expression, with *chitinase* transcripts increased throughout the analysed period and two *β-1,3-glucanase* isoforms mainly upregulated at 12 and 24 h after treatment (Wojtasik et al., 2024). In 14-days-old flax plants, application of Fo47 4 days before pathogen inoculation also enhanced the expression of genes encoding chitinase and β-1,3-glucanase. This response was accompanied by markedly reduced fusariosis symptoms, with the disease index decreased by approximately 30% at 28 days and by about 50% at 42 days after infection (Burgberger et al., 2026b). Together with similar observations in tomato and pepper, these findings indicate that Fo47-induced PR responses are not merely transient molecular changes but may contribute to sustained disease suppression, although their magnitude and timing depend on the host plant and experimental system. In flax, Fo47-mediated activation of PR-related responses has been associated with epigenetic regulation. Both pathogenic and non-pathogenic *Fusarium* strains were shown to alter DNA methylation patterns, including hypomethylation of resistance-related genes encoding β-1,3-glucanase and chitinase. Importantly, plants pretreated with Fo47 appeared to retain this hypomethylation pattern, which may support a faster or more efficient activation of these genes during subsequent pathogen infection (Wojtasik et al., 2019).

### 4.2 Reactive oxygen species and hypersensitive responses

Reactive oxygen species are produced rapidly after plants recognize microbial signals and represent one of the first cellular events associated with immune activation. In PTI, this oxidative burst is closely linked with other early signaling processes, including Ca^2 +^ influx and activation of MAPK cascades (de Lamo and Takken, 2020; Latz et al., 2018). Distinct ROS dynamics have been reported during interactions with pathogenic and non-pathogenic *F. oxysporum* strains. In flax cells, both the non-pathogenic strain Fo47 and the pathogenic strain *F. oxysporum* f.sp. *lini* (Foln) induced an early oxidative burst shortly after inoculation. However, only Fo47 triggered a second, stronger ROS burst approximately 3 h later and caused more pronounced localized cell death than Foln, particularly around 14 h after infection (Olivain et al., 2003). A similar response was also observed in tomato cell cultures. In this system, Fo47 and a pathogenic *F. oxysporum* strain induced different changes in the actin cytoskeleton and cell death, indicating that plant cells respond differently to non-pathogenic and pathogenic strains (Humbert et al., 2015). In flax, Fo47-mediated protection was linked to activation of the antioxidant system. Root priming with Fo47 reduced Foln colonization and induced early ROS-related responses in roots, including increased *NADPH oxidase D* transcript levels and reduced superoxide anion accumulation. Moreover, Fo47-primed plants showed systemic activation of enzymatic and non-enzymatic antioxidant responses in shoots after pathogen challenge, suggesting that ROS metabolism contributes to Fo47-induced priming against pathogenic *F. oxysporum* (Burgberger et al., 2026b).

### 4.3 Cell wall reinforcement and structural defenses

Structural modifications of plant tissues represent another important component of Fo47-associated resistance. Endophytic colonization may induce cell wall thickening, papilla formation, callose deposition, lignification, and accumulation of phenolic compounds (de Lamo and Takken, 2020; Latz et al., 2018). Fo47-induced wall thickenings and cell wall coilings have been proposed as structural barriers limiting pathogen progression (Aimé et al., 2008; Olivain et al., 2006). In pea roots, fungal penetration attempts were frequently restricted by callose-containing papillae deposited at infection sites (Benhamou and Garand, 2001; de Lamo and Takken, 2020). Similar responses have been reported in cucumber, tomato, and flax systems involving Fo47 (Benhamou et al., 2002; de Lamo and Takken, 2020; Le Floch et al., 2009; Olivain et al., 2003).

Recent studies in flax further support the involvement of cell wall remodeling in Fo47-mediated plant defense. In flax seedlings treated only with Fo47, dynamic changes in major cell wall polymers were observed, including modifications in cellulose organization, reduced hemicellulose content at selected time points, changes in pectin fractions and methylesterification, and a late increase in lignin content. These results suggest that Fo47 alone is sufficient to induce early structural reorganization of the flax cell wall, which may contribute to plant sensitization before pathogen attack (Wojtasik et al., 2024). Similar Fo47-induced changes were also reported in flax suspension cultures, where treatment with the non-pathogenic strain increased cellulose crystallinity and strengthened interactions between cell wall polymers, although lignin and pectin levels were lower than in untreated controls (Wróbel-Kwiatkowska et al., 2024). In whole flax plants exposed to pathogenic fungi, Fo47 pretreatment was associated with stronger structural defense responses. Plants treated with Fo47 and subsequently infected with *F. culmorum* showed higher FTIR signals characteristic of lignin and pectin, as well as increased cellulose crystallinity, compared with plants infected only with the pathogenic strain (Liszka et al., 2025). Together, these findings indicate that Fo47-induced cell wall remodeling is dynamic rather than uniformly reinforcing and that its structural outcome depends on the biological organization of the experimental system and the presence of pathogen challenge. Martínez-Soto et al. (2023) observed stronger callose deposition in the mature zone of primary roots and stronger lignin accumulation at the junctions between primary and lateral roots during Fo47 colonization than during pathogenic interactions in both *A. thaliana* and tomato. The same study reported stronger induction of *A. thaliana* genes involved in callose and lignin biosynthesis during Fo47 colonization compared with pathogenic *F. oxysporum* interactions. These differences may reflect both stronger host-mediated reinforcement of cell walls during Fo47 colonization and the lower capacity of Fo47 to degrade these structures compared with pathogenic strains (Martínez-Soto et al., 2023).

### 4.4 Plant primary and secondary metabolites

#### 4.4.1 Secondary metabolites

Secondary metabolites represent an important component of plant defense responses activated during interactions with beneficial endophytes. Endophytic microorganisms may stimulate plant secondary metabolism, including the production of antimicrobial compounds and metabolites involved in cell wall reinforcement (Fadiji and Babalola, 2020; Xia et al., 2022). Fo47 colonization has been linked with changes in phenolic metabolism. In pepper roots, Fo47 treatment was associated with the accumulation of caffeic acid, whereas in tomato roots, increased levels of ferulic acid were reported. These compounds may contribute to plant protection both through direct antimicrobial activity and by supporting structural reinforcement of the cell wall (de Lamo and Takken, 2020; Panina et al., 2007; Veloso et al., 2016). Thus, phenolic compounds may represent an important link between biochemical defense and physical restriction of pathogen progression. Recent studies in flax further support the role of Fo47 in the modulation of secondary metabolism. In flax suspension cultures, Fo47 was shown to act as an elicitor, increasing the total phenolic and flavonoid content and affecting cellular structural properties. These observations suggest that Fo47 alone can stimulate secondary metabolite accumulation even in the absence of pathogen challenge (Wróbel-Kwiatkowska et al., 2024). In whole flax plants, Fo47 pretreatment enhanced resistance to several pathogens. In plants pretreated with Fo47 and subsequently infected with *Fusarium culmorum*, increased levels of phenolic compounds and photosynthetic pigments were observed, together with FTIR changes indicating a higher contribution of lignin and pectin. This suggests that Fo47-induced phenolic metabolism may be associated with both enhanced antimicrobial capacity and reinforcement of structural defense barriers during pathogen infection (Liszka et al., 2025).

#### 4.4.2 Polyamines

Polyamines have also been implicated in plant protection mediated by beneficial microorganisms. Wheat seedlings colonized by the endophytic fungus *Piriformospora indica* displayed enhanced protection against *F. pseudograminearum* when treated with spermidine (Dehghanpour-Farashah et al., 2019). Likewise, suppression of *Macrophomina phaseolina* in soybean by *Exiguobacterium* spp. has been associated with the production of putrescine and spermine, which inhibited pathogen growth (Santos et al., 2021). Despite these observations, the contribution of polyamine metabolism to disease protection mediated by non-pathogenic *F. oxysporum* strains remains largely unexplored and warrants further research, as polyamines represent a promising but still poorly understood component of plant responses to Fo47. In flax seedlings, treatment with pathogenic and non-pathogenic *Fusarium* strains altered the expression of genes involved in polyamine biosynthesis and degradation, together with changes in putrescine, spermidine and spermine levels. However, pathogenic strains generally induced stronger increases in total and cell wall-bound polyamines than Fo47, suggesting that intensive polyamine accumulation is more closely associated with disease progression than with a balanced endophytic interaction (Wojtasik et al., 2015). More recent results indicate that effective priming by Fo47 is linked with reduced putrescine levels. Fo47 treatment decreased putrescine content in flax, whereas sustained elevation of putrescine promoted infection. After challenge with the pathogenic Foln strain, Fo47-primed plants showed early changes in the expression of polyamine metabolism-related genes, including arginine decarboxylase (adc), ornithine decarboxylase (odc) and diamine oxidase (dao), but maintained lower putrescine levels in roots and shoots than plants infected with Foln alone or co-treated with Fo47 and Foln. These observations suggest that controlled reduction of putrescine, rather than its persistent accumulation, may be an important component of Fo47-induced priming and restriction of pathogenic *F. oxysporum* development (Burgberger et al., 2026a).

### 4.5 Hormones

Hormonal signaling is an important regulatory layer of plant defense responses induced by beneficial endophytes, although Fo47-mediated resistance cannot be simply assigned to one classical hormonal pathway. In general, salicylic acid (SA) is mainly associated with systemic acquired resistance (SAR), whereas jasmonic acid (JA) and ethylene (ET) are commonly linked with induced systemic resistance (ISR). However, strong crosstalk between these pathways allows plants to adjust their response to a particular microbial interaction (Fadiji and Babalola, 2020; Latz et al., 2018; Pieterse et al., 2014; Xia et al., 2022). In the case of Fo47, studies in tomato indicate that endophyte-mediated resistance (EMR) differs from classical ISR and SAR, as protection against *F. oxysporum* f. sp. *lycopersici* was maintained in plants impaired in ET, JA or SA pathways (Constantin et al., 2019; de Lamo and Takken, 2020). This suggests that Fo47-induced EMR is not strictly dependent on the canonical SA-, JA- or ET-mediated defense routes. However, hormonal responses to Fo47 appear to be host dependent. In pepper, Fo47 treatment was associated with changes in defense-related hormonal compounds, including 12-oxo-phytodienoic acid (OPDA), SA and jasmonyl isoleucine (JA-Ile), and ethylene signalling contributed to protection against *V. dahliae* (Veloso et al., 2016; Veloso and Díaz, 2021). Therefore, Fo47 may influence hormone-associated defense networks, but the contribution of individual hormones depends on the plant species and pathosystem.

These examples show that Fo47-mediated plant protection cannot be attributed to a single defense pathway. Instead, Fo47 appears to modulate multiple and partially overlapping responses, including PR protein activation, ROS and antioxidant-system regulation, cell wall reinforcement or remodeling, secondary metabolism and hormone-associated signaling. Importantly, the contribution of these mechanisms differs between plant species and experimental systems, indicating that Fo47-induced resistance is strongly host- and context-dependent. Representative examples of plant responses associated with Fo47 colonization in different systems are summarized in Table 1.

**TABLE 1:** Plant responses and defense mechanisms associated with Fo47-mediated protection in different plant systems.

Plant/system	Response/mechanism	References
*Arabidopsis thaliana*	Enhanced expression of genes involved in callose and lignin biosynthesis	Martínez-Soto et al., 2023
	Enhanced callose deposition and lignin accumulation	
Tomato	Enhanced callose deposition and lignin accumulation	
Cucumber	Formation of wall appositions	Benhamou et al., 2002
	Deposition of dense/osmiophilic material along host cell walls and around invading hyphae	
	Occlusion of intercellular spaces with fibrillar/amorphous matrices	
Flax suspension cultures	Cell wall remodeling	Wróbel-Kwiatkowska et al., 2024
	Secondary metabolism elicitation	
Flax cell cultures	Second H_2_O_2_ burst	Olivain et al., 2003
	Stronger Ca^2 +^ influx	
	Higher percentage of localized cell death	
Flax	More frequent wall appositions	Burgberger et al., 2026a,b
	PR gene activation – *chitinase*, *beta-glucanase*	
	ROS metabolism and antioxidant-system induction	Burgberger et al., 2026b
	Cell wall reinforcement	Liszka et al., 2025
	Modulation of phenolic metabolism	
	Reduced putrescine levels	Burgberger et al., 2026a
Flax seedlings	PR gene activation	Wojtasik et al., 2024
	Cell wall remodeling	
	Epigenetic regulation of PR genes	Wojtasik et al., 2019
	Altered expression of genes involved in polyamine biosynthesis and degradation + changes in polyamines levels	Wojtasik et al., 2015
Pea roots	Callose-containing papillae deposited at infection sites	Benhamou and Garand, 2001
Pepper	Defense-related gene activation	Veloso and Díaz, 2012
	Hormone-associated defense responses	Veloso et al., 2016; Veloso and Díaz, 2021
	Modulation of phenolic metabolism	Veloso et al., 2016
Tomato	Modulation of phenolic metabolism	Panina et al., 2007
	Accumulation of PR proteins and defense enzymes	Fuchs et al., 1997
	PR protein accumulation	Duijff et al., 1998
	Priming of PR gene expression	Aimé et al., 2013
Tomato cell cultures	Cytoskeleton reorganization	Humbert et al., 2015
	Induction of localized cell death	

## 5 Fo47-mediated resistance mechanisms

Fo47-mediated disease protection is considered to result from the combined action of direct and indirect mechanisms. Direct mechanisms mainly include competition for nutrients and infection sites, inhibition of pathogen growth by antimicrobial compounds, and, in some systems, physical interaction with the pathogen or mycoparasitism (de Lamo et al., 2020; de Lamo and Takken, 2020; Fravel et al., 2003). As summarized in Figure 2, Fo47-mediated disease suppression involves both direct pathogen-targeting mechanisms and indirect plant-mediated responses, including defense priming and modulation of host immunity.

**FIGURE 2:**
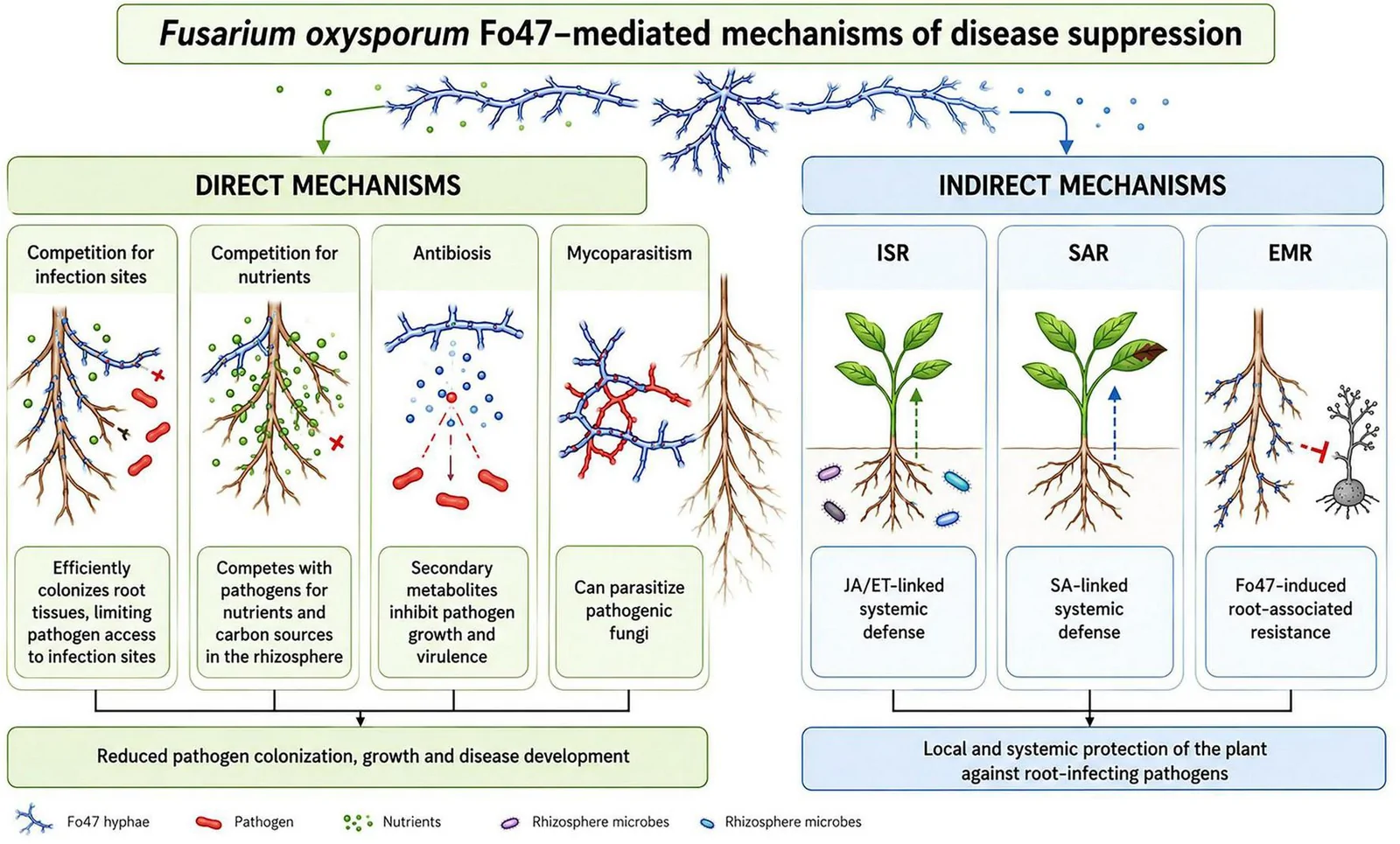
Schematic overview of the direct and indirect mechanisms by which *Fusarium oxysporum* Fo47 suppresses plant diseases. Direct mechanisms comprise competition for infection sites and nutrients, antibiosis, and mycoparasitism, whereas indirect mechanisms involve activation of ISR, SAR, and EMR. Together, these processes reduce pathogen colonization and disease development while enhancing local and systemic plant resistance.

### 5.1 Direct mechanisms of disease suppression

Fo47 can suppress soil-borne pathogens through direct interactions that limit their establishment, growth and development in the rhizosphere and root tissues. These mechanisms include competition for nutrients and root niches, antibiosis and direct inhibition of pathogen growth, as well as physical interference and potential mycoparasitism. Examples of Fo47-mediated biocontrol mechanisms and treatment strategies reported in different plant-pathogen systems are summarized in Table 2.

**TABLE 2:** Fo47-mediated biocontrol mechanisms and treatment strategies in different plant-pathogen systems.

Plant	Pathogen	Mechanism	Treatment type	References
Asparagus	*F. oxysporum* f. sp. *asparagi*	Competition for nutriens	Soil pre-colonization	Blok et al., 1997
Cucumber	*P. ultimum*	Antibiosis, mycoparasitism + induced resistance	Root priming	Benhamou et al., 2002
Eucalyptus	*F. oxysporum* strain Foeu1	Competition for niches	Root co-inoculation	Salerno et al., 2000
Flax	*F. oxysporum* f. sp. *lini* Foln3GUS	Competition for niches and nutrients	Root co-inoculation	Duijff et al., 1999
	*F. oxysporum* f. sp. *lini*	Competition for niches + induced local and systemic resistance	Root priming; root co-inoculation	Burgberger et al., 2026b
Pepper	*V. dahliae*, *P. capsici*	Antagonism/competition + induced resistance	Root priming	Veloso and Díaz, 2012
	*V. dahliae*	Competition for nutrients + induced resistance	Root priming	Veloso et al., 2016
Tomato	*F. oxysporum* f. sp. *lycopersici*	Induced resistance	Soil colonization; split root system	Fuchs et al., 1997
		Systemic induced resistance + direct antagonism	Root priming; split root system	Duijff et al., 1998
		Endophyte-mediated resistance (EMR)	Root co-inoculation	Constantin et al., 2019
				de Lamo et al., 2020
Tomato and pepper	*V. dahliae*	Host-dependent Fo47-induced resistance	Root priming	Veloso and Díaz, 2021

#### 5.1.1 Competition for nutrients and root niches

Competition for nutrients and root niches is one of the best-supported direct mechanisms associated with non-pathogenic *F. oxysporum* strains. Efficient colonization of the rhizosphere and root tissues allows Fo47 to occupy ecological niches that could otherwise be used by pathogenic strains. This may restrict pathogen access to infection sites and to available resources, particularly carbon compounds released by roots (Fravel et al., 2003; Latz et al., 2018; Lemanceau et al., 1993; Xia et al., 2022). Fo47 mediated protection through competition has been demonstrated in several plant-pathogen systems, including asparagus against *F. oxysporum* f. sp. *asparagi* (Blok et al., 1997), eucalyptus against *F. oxysporum* strain Foeu1 (Salerno et al., 2000), flax against *F. oxysporum* f. sp. *lini* (Duijff et al., 1999), and pepper against *V. dahliae* (Veloso et al., 2016). Fo47 reduced the population density and metabolic activity of *F. oxysporum* f. sp. *lini* Foln3GUS on the flax root surface, and the level of protection depended on the ratio between the non-pathogenic and pathogenic strains. The addition of *Pseudomonas putida* WCS358 further increased disease suppression, supporting the view that competition for both root niches and nutrients may limit pathogen establishment (Duijff et al., 1999).

#### 5.1.2 Antibiosis, growth inhibition and mycoparasitism

In addition to competition for nutrients and colonization sites, Fo47 may directly interfere with other microorganisms through growth inhibition, antibiosis and, in some interactions, mycoparasitism. Antibiosis generally involves the production of bioactive compounds that impair pathogen growth or metabolism (Minerdi et al., 2009; Raymaekers et al., 2020), whereas mycoparasitism involves direct physical contact with, penetration of, and exploitation of the target hyphae (Benhamou et al., 2002; Le Floch et al., 2009). Although these mechanisms are well recognized among microbial biocontrol agents, their contribution to Fo47-mediated protection appears to be highly dependent on the microorganism involved.

Detailed evidence for both antibiosis and mycoparasitism by Fo47 was obtained from its interaction with *P. ultimum*. In dual-culture assays, Fo47 substantially reduced *P. ultimum* growth, while pathogen cells collected from the interaction zone showed severe damage, including disorganization of the cytoplasm, loss of organelles and, after 3–4 days, extensive loss of cellular contents. The appearance of an inhibition halo around the pathogen colony further suggested the production of inhibitory compounds by Fo47. Mycoparasitism was particularly evident *in planta* rather than in dual culture: Fo47 hyphae were detected within extensively damaged and nearly empty *P. ultimum* cells both at the root surface and within infected cucumber root tissues. These findings demonstrated that Fo47 can suppress a pathogen through a combination of antibiosis and mycoparasitism while simultaneously stimulating plant defense responses (Benhamou et al., 2002). Similar Fo47-mediated antagonistic interactions were subsequently observed with the biocontrol oomycete *P. oligandrum*. In Petri-dish assays and in the tomato rhizosphere, Fo47 caused pronounced damage to *P. oligandrum* cells, and microscopic observations provided evidence for both antibiosis and mycoparasitism (Le Floch et al., 2009).

However, direct antagonistic activity is not consistently observed against all pathogens. In an *in vitro* assay, *V. dahliae* colonies grown in the presence of Fo47 developed more slowly than control colonies, suggesting that antagonism or competition may contribute to growth suppression, whereas the growth of *P. capsici* was not affected under the same experimental conditions (Veloso and Díaz, 2012). Similarly, when Fo47 and *F. oxysporum* f. sp. *lycopersici* strain Fol4287 were grown on the same PDA plate to assess direct antagonism, neither a visible inhibition halo nor a reduction in mycelial growth was observed (Constantin et al., 2020).

Recent studies have expanded the range of pathogens against which Fo47 exhibits direct growth-inhibitory activity. In dual-culture assays, Fo47 inhibited the growth of *Rhizoctonia solani* and *F. culmorum*, with maximum inhibition values of approximately 45% and 22%, respectively (Wróbel-Kwiatkowska et al., 2024). Fo47 was also shown to inhibit pathogenic *F. oxysporum*, with growth inhibition reaching 28% after 7 days and 25% after 10 days of dual culture (Liszka et al., 2025). These studies demonstrate direct antagonistic activity of Fo47, although the mechanisms responsible for the observed growth inhibition were not determined.

Available evidence demonstrates that Fo47 is capable of directly antagonizing selected fungal and oomycete microorganisms, although the nature and magnitude of these interactions vary considerably depending on the interacting organism and experimental conditions. Direct antagonism may therefore contribute to Fo47-mediated biocontrol, but its occurrence and relative importance appear to be interaction-dependent, while the mechanisms underlying the growth inhibition observed in more recent studies remain to be elucidated.

### 5.2 Indirect mechanisms of disease suppression

Besides direct antagonistic effects, Fo47-mediated disease suppression also involves indirect mechanisms based on the activation or modulation of plant defense responses. In plant pathology, induced resistance is commonly divided into SAR and ISR. However, beneficial microorganisms may activate defense responses that do not fully fit these classical categories, especially when resistance is induced locally in roots and is directed mainly against soil-borne pathogens (de Lamo and Takken, 2020; Latz et al., 2018; Xia et al., 2022).

#### 5.2.1 Endophyte-mediated resistance and its relationship with ISR and SAR

More recent studies have refined this concept and proposed that Fo47-induced resistance is better described as EMR, which is a form of resistance induced by endophytic or non-host *F. oxysporum* strains and is considered distinct from classical ISR and SAR. In tomato, Fo47-mediated protection against *F. oxysporum* f. sp. *lycopersici* was maintained in plants impaired in ET, JA or SA signaling pathways. This indicates that Fo47-induced EMR is not strictly dependent on the canonical hormonal pathways usually associated with ISR or SAR (Constantin et al., 2019; de Lamo et al., 2020; de Lamo and Takken, 2020). Additional evidence also suggests that Fo47-mediated EMR differs from other forms of induced resistance. Fo47 provided stronger protection against tomato wilt disease than avirulent *F. oxysporum* strains used for cross-protection, suggesting that its protective effect is not simply based on classical R-gene-mediated recognition of avirulent pathogen effectors. Moreover, PTI was shown to restrict colonization by endophytic *Fusarium* strains, but it did not abolish Fo47-mediated protection, indicating that EMR can operate even when early immune perception affects endophyte colonization (de Lamo et al., 2020; de Lamo and Takken, 2020). These results support the view that Fo47-induced resistance involves a specific plant-endophyte interaction rather than a simple activation of general defense pathways.

Overall, ISR and SAR provide useful background for understanding induced resistance, but Fo47-mediated protection is more accurately described as EMR. This mechanism combines local root colonization by a non-pathogenic endophyte with plant-mediated defense activation, and it is often independent of the classical SA-, JA- and ET-dependent pathways. Therefore, Fo47-mediated disease suppression should be viewed as a complex process in which direct antagonism, root niche occupation and EMR act together to reduce pathogen colonization and disease development.

#### 5.2.2 Host-, pathogen- and treatment-dependent variability of Fo47-induced protection

A characteristic feature of Fo47-mediated EMR is its strong association with root protection. Fo47 has been reported to suppress several root-infecting pathogens, whereas protection against foliar pathogens is limited or absent. For example, in pepper, Fo47 reduced disease symptoms caused by *V. dahliae* and *P. capsici* but did not protect against the foliar pathogen *B. cinerea* (de Lamo and Takken, 2020; Veloso and Díaz, 2012). The response induced by Fo47 may also vary between host plants. In closely related Solanaceae species, Fo47 triggered distinct defense outputs in tomato and pepper, including differences in hormone-related responses and protection efficiency against *V. dahliae* (Veloso and Díaz, 2021). This indicates that Fo47-induced resistance should not be considered a fixed mechanism acting identically in all plants. Rather, EMR appears to be a flexible defense state shaped by the host, the pathogen and the timing of endophyte colonization.

#### 5.2.3 Fo47-induced priming

Priming is a physiological state in which plants acquire an enhanced capacity to activate defense responses after subsequent pathogen attack. Unlike constitutive defense activation, primed plants do not continuously maintain high levels of defense-related activity. Instead, they remain in a state of increased readiness, which allows faster and more effective responses when they are challenged by pathogens. In the context of biological control, this strategy is particularly advantageous because it may improve resistance while reducing the metabolic costs associated with permanent activation of immune pathways (Aimé et al., 2008; Conrath et al., 2002). Several observations indicate that priming contributes to Fo47-mediated disease protection. Disease suppression is often more effective when Fo47 is applied before pathogen exposure than when beneficial and pathogenic strains are introduced simultaneously. This temporal requirement suggests that Fo47-mediated protection involves a preparatory phase during which physiological, molecular and structural changes are established before pathogen challenge occurs. Early studies in tomato showed that Fo47 reduced disease caused by *F. oxysporum* f. sp. *lycopersici* even when the beneficial and pathogenic strains were separated spatially or temporally, indicating that protection could not be explained solely by direct microbial antagonism (Fuchs et al., 1997). Further evidence was obtained in tomato roots pre-inoculated with Fo47 and subsequently challenged with Fol8, where genes encoding chitinase, β-1,3-glucanase and PR-1a were overexpressed, while pathogen growth in roots was reduced. These results support the view that Fo47 can prepare host tissues for a more efficient defense response after pathogen attack (Aimé et al., 2013). In many Fo47-related systems, disease suppression occurs without strong constitutive activation of defense responses following endophyte colonization alone. Fo47-treated plants may display lower expression of defense-related genes than pathogen-infected plants, while still showing enhanced resistance to subsequent infection (Aimé et al., 2008; Conrath et al., 2002). This indicates that Fo47-mediated protection may involve a latent state of defense readiness, in which immune responses are activated more rapidly or more strongly only after pathogen challenge. Moreover, Fo47-induced modifications of root tissues, including cell wall thickenings and cell wall coilings, have been proposed to contribute to a primed state that improves the plant’s capacity to restrict pathogen invasion (Aimé et al., 2008; Olivain et al., 2006). In flax, Fo47-induced priming may involve both epigenetic and structural preparation of plant tissues. Fo47 pretreatment was associated with hypomethylation of resistance-related genes encoding β-1,3-glucanase and chitinase, suggesting that epigenetic memory may facilitate faster activation of PR-related genes during subsequent infection by pathogenic *F. oxysporum* (Wojtasik et al., 2019). More recent work also showed that Fo47 alone can induce defense-related changes in flax seedlings, including activation of PR-related genes and remodeling of cell wall components. This suggests that Fo47 may sensitize flax not only at the level of defense gene expression, but also through structural preparation of the cell wall before pathogen attack (Wojtasik et al., 2024). Our recent studies on 14-days-old flax plants support the importance of timing in Fo47-mediated priming. When Fo47 was applied 4 days before inoculation with the pathogenic Foln strain, disease development and pathogen colonization were strongly reduced. In contrast, simultaneous application of Fo47 and Foln was much less effective and did not provide comparable protection. This indicates that prior establishment of the non-pathogenic strain is necessary for efficient suppression of pathogen development and supports the concept that Fo47 requires time to induce a protective state in the host plant (Burgberger et al., 2026a,b). Recent studies on flax further support the protective value of Fo47 pretreatment, as pretreated plants showed increased resistance to several pathogens, including *F. oxysporum*, *F. culmorum* and *R. solani* (Liszka et al., 2025). The priming effect observed in flax involved both local and systemic responses. In roots, Fo47 induced early *chitinase* and *NADPH oxidase D* gene transcript accumulation and reduced superoxide anion levels, suggesting activation of ROS-related defense signaling. After Foln challenge, primed plants also showed systemic activation of enzymatic and non-enzymatic antioxidant responses in shoots, including changes in ROS metabolism-related genes, antioxidant enzyme activity, phenolic compounds and antioxidant potential. These results indicate that Fo47 prepares flax plants for a coordinated response to infection, involving PR gene activation, ROS regulation, antioxidant defense, metabolic changes and restriction of pathogen colonization (Burgberger et al., 2026b).

Current evidence indicates that Fo47-mediated protection is most effective when the non-pathogenic strain is established before pathogen infection. This supports the view that Fo47 acts as a priming agent rather than only as a direct antagonist. Fo47-induced priming should therefore be understood as a complex defense state involving molecular, biochemical, metabolic and structural changes that prepare the plant for a faster and more effective response to subsequent pathogen attack.

### 5.3 Integration of direct and indirect mechanisms

Competition for nutrients and root niches appears to be a central direct mechanism of Fo47-mediated disease suppression, especially when Fo47 establishes in the root environment before pathogen arrival. However, competition alone is unlikely to fully explain Fo47-mediated protection under all conditions, particularly under high pathogen pressure. Instead, direct mechanisms such as niche occupation, resource competition, antibiosis and mycoparasitism probably act together with indirect plant-mediated responses, including endophyte-mediated resistance and priming (de Lamo and Takken, 2020; Fravel et al., 2003; Lahlali et al., 2014; Latz et al., 2018; Xia et al., 2022).

### 5.4 Alternative microbial biocontrol systems against pathogens suppressed by Fo47

A range of alternative microbial biocontrol systems has also been reported for the soil-borne pathogens discussed above, including *F. oxysporum* f. sp. *asparagi*, *P. ultimum*, *F. oxysporum* f. sp. *lini*, *P. capsici*, *F. oxysporum* f. sp. *lycopersici*, and *V. dahliae*. These systems involve antagonistic fungi, particularly *Trichoderma* spp., as well as beneficial bacteria belonging to genera such as *Bacillus* or *Pseudomonas*. Many of these bacteria are classified as plant growth-promoting rhizobacteria (PGPR), which colonize the rhizosphere and promote plant growth directly or indirectly, including through pathogen suppression (Bhattacharyya and Jha, 2012). As microbial biocontrol have recently been reviewed comprehensively (Anand and Yadav, 2026; Boro et al., 2022; Xiong et al., 2026), only selected systems relevant for comparison with Fo47 are presented here. Examples of these alternative biocontrol agents and their potential mechanisms of action are summarized in Table 3.

**TABLE 3:** Microbial biocontrol agents other than Fo47 and their potential mechanisms of action against selected soilborne plant pathogens.

Pathogen	Biocontrol agent	Plant	Potential mechanism of action	References
*Fusarium oxysporum* f. sp. *asparagi*	*Bacillus velezensis* FC37; alternatively, *Bacillus paralicheniformis* Hvs2	Asparagus	Production of hydrolytic enzymes; antifungal volatile compounds	Lastra et al., 2021
	*Trichoderma atroviride*, *T. asperellum*, and *T. saturnisporum*		Mechanism not determined	Brizuela et al., 2023
*Pythium ultimum*	*Trichoderma harzianum*	Lettuce	Effective rhizosphere colonization and suppression of pathogen propagules	Lynch et al., 1991
	*Trichoderma virens* G-93 or GL-21	*Catharanthus roseus* (vinca)	Antibiosis potentially mediated by gliotoxin production	Burns and Benson, 2000
	*Pseudomonas rhodesiae* HAI-0804	Cucumber	Efficient root colonization and biofilm formation, rather than antibiotic activity or phytohormone-dependent defense induction	Takeuchi et al., 2024
	*Pseudomonas* sp. IALR1619	Cucumber, lettuce	Antifungal activity mediated by volatile organic compounds	Amaradasa et al., 2024
*Fusarium oxysporum* f. sp. *lini*	*Bacillus subtilis* ATCC 6633	Flax	Plant cell wall remodeling and reinforcement	Planchon et al., 2021
	*Pseudomonas* spp. + Fo47		Directly through antibiosis and/or indirectly through iron competition	Mazurier et al., 2009
	*Trichoderma harzianum*		Mechanism not determined	Raghuvanshi et al., 2024
	Fo47 + *Pseudomonas putida* WCS358		Root-level competition and pseudobactin-mediated iron competition.	Duijff et al., 1999
	*Pseudomonas fluorescens*		Antimicrobial metabolite-mediated antagonism	Toua et al., 2013
*Phytophthora capsici*	*Trichoderma asperellum* T34	Pepper	Competition and mycoparasitism, with possible inhibition of zoospore germination	Segarra et al., 2013
	*Bacillus subtilis* ME488		Production of antibiotics	Chung et al., 2008
	*Bacillus velezensis* H12, *B. safensis* H23		Hydrolytic enzyme production, induction of PR1 and PR4 expression	Fu et al., 2025
*Fusarium oxysporum* f. sp. *lycopersici*	*Trichoderma asperellum* T34	Tomato	Competition for iron	Segarra et al., 2010
	*Paenibacillus polymyxa* BHUPSB16, *Bacillus cereus* IESDJP-V4		Induction of antioxidant and defense-related responses	Ansari et al., 2025
	*Trichoderma harzianum* AMUTH-1 *and T. asperellum* AMUTV-3		Hydrolytic enzyme production and siderophore-mediated competition.	Haque et al., 2023
*Verticillium dahliae*	*Bacillus subtilis* NCD-2	Cotton	Induction of systemic resistance through SA- and JA-dependent signaling	Mo et al., 2025
	*Bacillus subtilis* KRS015		Antifungal VOCs production and induction of ROS- and defense-related responses	Song et al., 2024
	*Bacillus altitudinis* strain KRS010		Antifungal VOCs and extracellular metabolites, inhibition of melanin biosynthesis, SA/JA-dependent ISR	Shan et al., 2024

## 6 Horizontal transfer of pathogenicity determinants as a potential limitation of endophytic biocontrol agents

While the preceding sections focused on the mechanisms underlying Fo47-mediated disease suppression, the potential use of Fo47 and related endophytic strains as biological control agents must also be considered from a long-term biosafety perspective. In particular, the genomic plasticity of *F. oxysporum* and the mobility of accessory chromosomes raise the possibility that non-pathogenic strains may acquire pathogenicity-associated genetic material. The following section therefore shifts from the mechanisms of plant protection to the potential genomic risks associated with the agricultural application of endophytic *F. oxysporum* strains.

Horizontal gene transfer (HGT) is a widespread evolutionary process involving the exchange of genetic material between organisms outside of parent-offspring inheritance (Steele et al., 2019). While HGT has been extensively documented in prokaryotes, increasing evidence indicates that it also contributes significantly to fungal evolution, adaptation, and pathogenicity (Ciach et al., 2024; Liu et al., 2025). Because beneficial and pathogenic microorganisms frequently coexist within the same ecological niches, understanding the frequency and consequences of HGT is essential when evaluating the long-term safety of microbial biocontrol strategies.

The genome of *F. oxysporum* is composed of a conserved core genome and a highly variable accessory compartment, also referred to as lineage-specific (LS) or supernumerary regions, which differ in gene content, density, and evolutionary dynamics. While the core genome remains relatively stable among members of the *F. oxysporum* species complex, the accessory genome is considered the primary determinant of host adaptation and pathogenicity (Armitage et al., 2018; Ma et al., 2010; Samsonova et al., 2021). In several plant pathogenic fungi, virulence-associated genes are concentrated on dispensable supernumerary chromosomes. In *F. oxysporum*, pathogenicity-related chromosomes have been identified in strains infecting tomato and cucurbit hosts (Li et al., 2020a,b). The presence of these lineage-specific chromosomes is strongly associated with the ability to cause disease (Ma et al., 2010). Conversely, loss of pathogenicity chromosomes results in loss of virulence (Vlaardingerbroek et al., 2016). Pathogenicity chromosomes contain virulence-associated genes, including *SIX* effectors involved in host colonization (de Lamo and Takken, 2020; Schmidt et al., 2013). Importantly, these accessory chromosomes can be horizontally transferred between strains, and even partial chromosome transfer may be sufficient to convert a non-pathogenic isolate into a pathogen (Selin et al., 2016; van der Does and Rep, 2012). Some of the experimentally confirmed HGT events in fungi are presented in Table 4. The best-known example involves the interaction between the non-pathogenic strain Fo47 and *F. oxysporum* f. sp. *lycopersici*. In this system, horizontal transfer of lineage-specific chromosomes, including chromosome 14 carrying several *SIX* genes, converted Fo47 into a tomato pathogen capable of causing vascular wilt (de Lamo and Takken, 2020; Ma et al., 2010). Similar observations were later reported for pathogenicity chromosomes originating from cucurbit-infecting strains, which also transformed Fo47 into a pathogen of cucurbit hosts (van Dam et al., 2017b). These findings demonstrate that acquisition of accessory pathogenicity chromosomes can rapidly generate new virulent lineages without requiring long-term evolutionary adaptation.

**TABLE 4:** Experimentally confirmed HGT events in fungi.

Species	Transferred gene	References
*Fusarium oxysporum*	SIX1/chromosome 14	Ma et al., 2010
	Chromosome RC	van Dam et al., 2017b
	SIX6/chromosome RC	Li et al., 2020b
	Pathogenicity chromosome	Li et al., 2020a
*Aspergillus fumigatus*	Cyp51A/triazole resistance	Morogovsky et al., 2022
*Metarhizium robertsii*	Chromosome A	Habig et al., 2024

The capacity of fungi to acquire biologically relevant traits through horizontal transfer is not limited to *F. oxysporum*. One of the earliest documented examples involved the transfer of the virulence gene *ToxA* from *Parastagonospora nodorum* to *Pyrenophora tritici-repentis*, leading to the emergence of a new wheat pathogen (Friesen et al., 2006). More recently, numerous HGT events have been identified in fungal genomes, including 134 putative transfers in species of *Armillaria* associated with virulence and biomass degradation (Sahu et al., 2023), 829 foreign proteins detected in early-diverging fungi (Ciach et al., 2024), and 8,815 independent prokaryote-to-fungus gene acquisition events identified through comparative analysis of 829 fungal genomes (Liu et al., 2025). The propensity of *F. oxysporum* for genetic exchange may be further facilitated by the abundance of transposable elements within its genome. Notably, transposable elements constitute approximately 3.98% of the genome of *F. oxysporum* f. sp. *lycopersici* 4287, compared with only 0.14% in *F. verticillioides* and 0.03% in *F. graminearum* (Daboussi and Langin, 1994; Ma et al., 2010). Such genomic plasticity is thought to contribute to the remarkable adaptability and evolutionary potential of the species complex.

From the perspective of biological control, these observations highlight the importance of evaluating the likelihood of horizontal transfer events under agricultural conditions. Although HGT has been demonstrated under experimental settings, its frequency and ecological significance in natural plant-associated environments remain poorly understood. Acquisition of mobile chromosomes does not necessarily confer pathogenicity. In horizontal chromosome transfer (HCT) experiments, the non-pathogenic cabbage-associated *F. oxysporum* strain 08C-3B acquired mobile chromosomes from the pathogenic *F. oxysporum* f. sp. *conglutinans* strain, but the resulting progeny did not become pathogenic on cabbage (Ayukawa et al., 2026). This example demonstrates that the occurrence of chromosome transfer should be distinguished from its phenotypic consequences. Therefore, assessment of chromosome transfer frequencies between beneficial and pathogenic *F. oxysporum* strains remains essential for determining the long-term biosafety of Fo47 and other endophytic strains intended for large-scale agricultural application (de Lamo and Takken, 2020).

## 7 Omics-based approaches for the detection and characterization of non-pathogenic *F. oxysporum* strains

Omics-based approaches provide complementary layers of information that can support the detection, discrimination, and functional characterization of non-pathogenic *F. oxysporum* strains by integrating genomic, transcriptomic, proteomic, and metabolomic data. Whole-genome sequencing enables comparisons of the core and accessory genomes, pathogenicity-associated chromosomes, *SIX* genes and other candidate effectors, *mimp* elements located near effector genes, genes encoding cell wall-degrading enzymes, transposable elements, and secondary metabolite clusters (Constantin et al., 2021; Ma et al., 2010; Martínez-Soto et al., 2023). Comparative genomic analysis of endophytic and pathogenic *F. oxysporum* isolates revealed that endophytic strains generally contained fewer *mimp* elements and fewer candidate effector genes in their accessory genomes. Analysis of the presence and absence of 170 candidate effectors grouped 43 of the 45 examined endophytic isolates together. However, no single gene set was identified as being exclusive to endophytic strains. The distinction between endophytic and pathogenic isolates was therefore associated mainly with a reduced candidate effector repertoire rather than with the presence of universal endophyte-specific genes (Constantin et al., 2021). Van Dam et al. compared the genomes of 88 isolates and designed 18 markers based on candidate effector genes. These markers distinguished pathogenic formae speciales from other strains, including non-pathogenic isolates. The TaqMan assay enabled the detection of as little as 2.5 pg of DNA, and the authors indicated that the markers could be applied directly to DNA isolated from plants or soil (van Dam et al., 2017a). Together, these studies indicate that differences in candidate effector repertoires can distinguish most endophytic isolates from pathogenic lineages and support the development of specific molecular markers, although no universal gene set characteristic of endophytic strains has been identified.

The genome indicates which genes are present in a strain, whereas RNA sequencing reveals which genes are actively expressed during colonization. Dual RNA-seq or metatranscriptomics enables the simultaneous analysis of fungal and plant transcripts. Fo47 and the pathogenic strain Fo5176 share a core genome of approximately 46 Mb but contain 1,229 and 5,415 unique accessory genes, respectively. Metatranscriptomic comparison of *A. thaliana* interactions with Fo47 and the pathogenic strain Fo5176 showed that most plant transcriptional responses were common to both interactions. However, the pathogenic interaction induced stronger stress-related responses and suppressed growth-associated processes, whereas Fo47 attenuated parts of the immune response and stimulated nitrogen assimilation (Guo et al., 2021). These findings indicate that transcriptomics can provide a functional profile of endophytic colonization, although such profiles may depend on the host, tissue and sampling time.

Proteomic and secretomic analyses can detect secreted effectors, cell wall-degrading enzymes, fungal proteins present in the xylem, and characteristic host defense proteins (Gawehns et al., 2015; Roy et al., 2023; Zhao et al., 2020). In an analysis of the tomato xylem sap proteome, infection with the pathogenic strain Fol007 caused substantial changes in protein abundance. In contrast, treatment with Fo47 alone did not result in the detection of significantly differentially abundant proteins or Fo47 proteins in the xylem sap. During Fo47-induced resistance, characteristic changes in PR-5 isoforms were also observed, which distinguished endophyte-mediated resistance from resistance dependent on an R gene (de Lamo et al., 2018). Proteomics is therefore more suitable for characterizing the molecular course of an interaction than for identifying the fungal isolate itself.

Metabolomics can identify secondary metabolites, antimicrobial compounds, volatile organic compounds, and plant metabolites induced by an endophyte. Comparative analysis of volatile organic compound profiles revealed differences between pathogenic and non-pathogenic fungi. However, no compound was identified that occurred in all examined non-pathogenic fungi while being absent from pathogenic fungi (Moisan et al., 2019). These findings indicate that metabolomics can support fungal strain classification and the identification of potential biocontrol candidates but does not by itself provide definitive confirmation of non-pathogenicity.

## 8 Genome-editing technologies for functional characterization and improvement of non-pathogenic fungi

Genome-editing technologies provide powerful tools for investigating the biology of non-pathogenic fungi. CRISPR/Cas systems enable targeted disruption, insertion, replacement or tagging of selected genomic loci. In filamentous fungi, Cas9 and guide RNA can be delivered using integrating or transiently maintained vectors, or directly as preassembled Cas9-sgRNA ribonucleoprotein complexes (RNPs) (Shen et al., 2024; Song et al., 2019; Wang and Coleman, 2019).

One of the main applications of genome editing in non-pathogenic fungi is the functional validation of genes implicated in endophytic colonization and beneficial interactions with plants. Candidate genes identified through genomics, transcriptomics, proteomics or metabolomics can be disrupted or modified to determine whether they participate in plant tissue colonization, regulation of fungal growth within the host, modulation of plant immunity, competition with pathogens, mycoparasitism, stress tolerance or production of bioactive metabolites. For example, Huang et al. developed a CRISPR/Cas9 system for fungus *Phomopsis liquidambaris* and disrupted *PmkkA*, which encodes a mitogen-activated protein kinase associated with the cell wall integrity pathway. Rice seedlings colonized by the Δ*PmkkA* mutant showed stronger accumulation of reactive oxygen species and increased chitinase and glucanase activities compared with plants colonized by the wild-type strain. However, these enhanced defense responses were accompanied by suppression of plant growth. These findings indicated that *PmkkA* contributes to the maintenance of a balanced interaction between the endophyte and its host, including regulation of plant immune activation (Huang et al., 2020). Thus, targeted gene disruption can reveal mechanisms underlying endophytic compatibility, but deletion of a gene involved in immune suppression does not necessarily generate a more beneficial strain.

Genome editing may also increase the biosafety of beneficial fungi by removing genes responsible for the production of toxic or otherwise undesirable metabolites. This application is especially relevant to grass endophytes of the genus *Epichloë*. Although these fungi may improve plant persistence and resistance to herbivores or environmental stress, some strains produce ergot alkaloids that are toxic to grazing animals. Cas9 RNP-mediated editing was used in *Epichloë coenophiala* to remove two complete ergot alkaloid biosynthesis clusters. The EAS1 and EAS2 clusters, approximately 196 and 75 kb in length, respectively, were deleted either individually or simultaneously. The same approach also enabled the deletion of individual genes involved in alkaloid biosynthesis. The resulting strains lacked the targeted toxin-associated genes and did not contain a stably integrated Cas9 transgene, demonstrating that genome editing can remove undesirable traits while largely preserving the remaining genetic background of a beneficial endophyte (Florea et al., 2021).

Another potential application is the modification of fungal secondary metabolism. CRISPR/Cas systems can be used to delete pathway repressors, modify cluster-specific regulators, activate weakly expressed biosynthetic pathways or introduce genes involved in the production of antimicrobial and plant-beneficial metabolites. In *P. liquidambaris*, a modified CRISPR/Cas9 strategy using two guide RNAs was used for pathway refactoring and activation of flavonoid biosynthesis, including the production of naringenin and kaempferol (Yang et al., 2021). This example demonstrates that genome editing can alter the metabolic capacity of an endophytic fungus.

A versatile vector-based CRISPR/Cas9 system has also been developed for several *Fusarium* strains. Shinkado et al. demonstrated targeted knockout, knock-in, nucleotide substitution and endogenous gene tagging in *F. oxysporum*, including editing of genes located in accessory genomic regions. The same system was subsequently applied without vector modification to the non-pathogenic biocontrol strain *Fusarium commune* W5, which provides direct evidence that editing systems developed for pathogenic *Fusarium* strains can also function efficiently in a non-pathogenic biocontrol isolate (Shinkado et al., 2022).

Despite its potential, genome editing in non-pathogenic filamentous fungi remains technically challenging. Editing efficiency may be limited by low transformation rates, difficulties in protoplast generation and regeneration, dominance of non-homologous end joining over homologous recombination, heterokaryosis and the limited availability of suitable promoters and selectable markers. Off-target mutations, unexpected integration of vector fragments and pleiotropic effects of target-gene disruption must also be considered. In beneficial fungi, even an accurately introduced mutation may alter growth, sporulation, stress tolerance, host range, tissue colonization, competition with other microorganisms or production of non-target metabolites (Shen et al., 2024; Song et al., 2019; Verma et al., 2023; Wang and Coleman, 2019).

## 9 Conclusion

*Fusarium oxysporum* Fo47 is a well-characterized non-pathogenic, endophytic strain with strong potential as a biological control agent against root-associated pathogens. Its protective activity results from the combined action of direct mechanisms, such as competition for nutrients and infection sites, and indirect plant-mediated responses, including defense priming and endophyte-mediated resistance. Fo47 does not usually induce constant defense activation but rather prepares the plant for a faster and more effective response after pathogen challenge. This response may involve PR proteins, ROS and antioxidant metabolism, cell wall remodeling, secondary metabolites, polyamine regulation and hormone-associated signaling. However, the contribution of these mechanisms depends on the host plant, pathogen and timing of Fo47 application. At the same time, the use of Fo47 and other non-pathogenic *F. oxysporum* strains requires careful biosafety evaluation. The considerable genomic plasticity of the *F. oxysporum* species complex is illustrated by the relatively high proportion of transposable elements in the genome of *F. oxysporum* f. sp. *lycopersici* 4287, where they constitute approximately 3.98% of the genome, compared with only 0.14% in *F. verticillioides* and 0.03% in *F. graminearum*. Together with the ability of *F. oxysporum* strains to exchange accessory pathogenicity chromosomes, this genomic plasticity highlights the need to assess the frequency and phenotypic consequences of horizontal transfer under plant-associated and agricultural conditions. Therefore, Fo47 should be considered both a promising biocontrol agent and an important model for studying the balance between beneficial endophytic colonization, plant defense activation and fungal genome plasticity.
